# Automated Synthesis of ^68^Ga-Labeled DOTA-MGS8 and Preclinical Characterization of Cholecystokinin-2 Receptor Targeting

**DOI:** 10.3390/molecules27062034

**Published:** 2022-03-21

**Authors:** Anton Amadeus Hörmann, Elisabeth Plhak, Maximilian Klingler, Christine Rangger, Joachim Pfister, Gert Schwach, Herbert Kvaternik, Elisabeth von Guggenberg

**Affiliations:** 1 Department of Nuclear Medicine, Medical University of Innsbruck, 6020 Innsbruck, Austria; anton.hoermann@i-med.ac.at (A.A.H.); klinglermaximilian@gmail.com (M.K.); christine.rangger@i-med.ac.at (C.R.); joachim.pfister@i-med.ac.at (J.P.); 2 Department of Pharmaceutical Chemistry, Institute of Pharmaceutical Sciences, University of Graz, 8010 Graz, Austria; elisabeth.plhak@medunigraz.at; 3 Department of Radiology, Division of Nuclear Medicine, Medical University of Graz, 8036 Graz, Austria; herbert.kvaternik@medunigraz.at; 4 Division of Immunology and Pathophysiology, Otto Loewi Research Center, Medical University of Graz, 8010 Graz, Austria; gert.schwach@medunigraz.at

**Keywords:** minigastrin, automated synthesis, cholecystokinin-2 receptor, radiometals, molecular imaging

## Abstract

The new minigastrin analog DOTA-MGS8 targeting the cholecystokinin-2 receptor (CCK2R) used in this study displays the combination of two site-specific modifications within the C-terminal receptor binding sequence together with an additional N-terminal amino acid substitution preventing fast metabolic degradation. Within this study, the preparation of ^68^Ga-labeled DOTA-MGS8 was validated using an automated synthesis module, describing the specifications and analytical methods for quality control for possible clinical use. In addition, preclinical studies were carried out to characterize the targeting potential. [^68^Ga]Ga-DOTA-MGS8 showed a high receptor-specific cell internalization into AR42J rat pancreatic cells (~40%) with physiological expression of rat CCK2R as well as A431-CCK2R cells transfected to stably express human CCK2R (~47%). A favorable biodistribution profile was observed in BALB/c nude mice xenografted with A431-CCK2R cells and mock-transfected A431 cells as control. The high tumor uptake of ~27% IA/g together with low background activity and limited uptake in non-target tissue confirms the potential for high-sensitivity positron emission tomography of stabilized MG analogs in patients with MTC and other CCK2R-related malignancies.

## 1. Introduction

During the last decades, receptor-mediated tumor targeting using radiolabeled peptides has gained increasing attention in the field of nuclear medicine. The use of cell surface structures such as G-protein-coupled receptors (GPCRs) as biological targets has provided new possibilities for theranostic approaches. These receptors are overexpressed by various carcinomas in order to take advantage of the endogenic ligands for cell proliferation, angiogenesis and metastasis [1]. Radiolabeled derivatives of these endogenic neuropeptides can be used in the early detection and treatment of cancer.

The cholecystokinin-2 receptor (CCK2R) is one of these seven-transmembrane receptors, physiologically present in the stomach, pancreas and brain, but also overexpressed by various tumors [2,3,4]. This receptor is widely abundant in medullary thyroid carcinomas (MTCs), stromal ovarian cancers, astrocytomas, small-cell lung cancers as well as gastroenteropancreatic neuroendocrine tumors (GEP-NETs) and other tumor entities [5,6,7]. The feasibility of CCK2R targeting with radiolabeled peptides derived from the natural ligands minigastrin (MG) and cholecystokinin has already been proven for patients with advanced MTC as well as other CCK2R-related malignancies [8,9]. However, the critical problem of the radiolabeled ligands developed in the past is the very low in vivo stability of the linear peptide sequence when injected intravenously [10,11].

**Figure 1:**
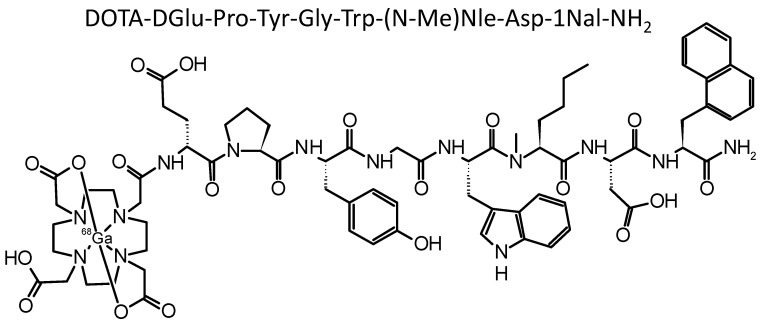
Amino acid sequence of DOTA-MGS8 and chemical structure of [^68^Ga]Ga-DOTA-MGS8.

In recent years, considerable progress has been made in the development of modified peptide analogs with increased stability and enhanced tumor targeting [12,13,14,15,16]. Different modifications were introduced in the amino acid sequence of the new peptide derivative DOTA-MGS8—two site-specific modifications within the C-terminal receptor binding sequence as well as insertion of proline in the N-terminal part of the peptide (for exact chemical structure, see Figure 1). In previous studies by our group, DOTA-MGS8 was proven to have promising advantages in terms of high in vivo stability and enhanced tumor uptake when radiolabeled with indium-111 and lutetium-177 [15,16].

To progress toward the clinical application of DOTA-MGS8 labeled with gallium-68 for high-sensitivity positron emission tomography (PET), we have validated the automated synthesis of this new radiopharmaceutical. A modified Scintomics GRP module connected to two Galli Ad ^68^Ge/^68^Ga generators was used for the automated radiolabeling process. The product specifications, including analytical procedures and acceptance criteria, were adopted from the Ph. Eur. monograph for gallium (^68^Ga) edotreotide injection (2482). Furthermore, preclinical in vitro and in vivo studies were performed to evaluate the targeting potential of [^68^Ga]Ga-DOTA-MGS8. Cell internalization studies were carried out in two different cell lines: AR42J rat pancreatic cells with physiological expression of rat CCK2R as well as A431-CCK2R cells transfected to stably express human CCK2R [17,18]. In addition, the biodistribution in BALB/c nude mice xenografted with A431-CCK2R cells and mock-transfected A431 cells as negative control were investigated to confirm the diagnostic properties in vivo.

## 2. Results

### 2.1. Radiolabeling and Quality Control

**Figure 2:**
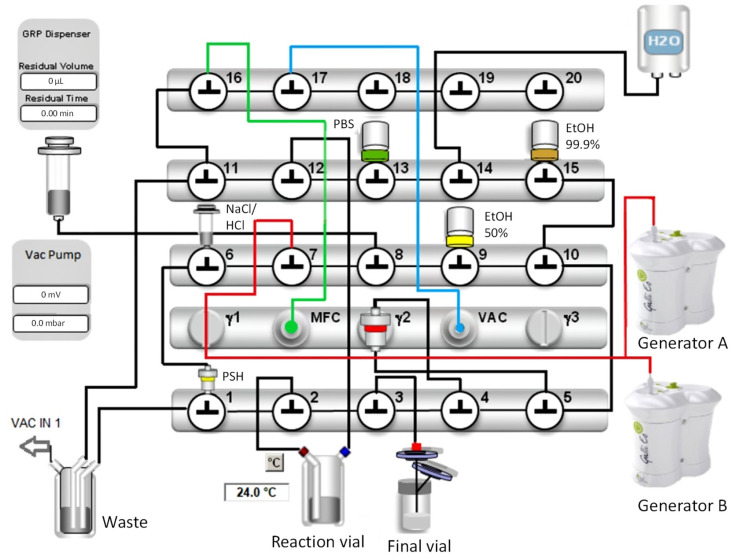
Synthesis scheme for the automated production of [^68^Ga]Ga-DOTA-MGS8 using the described configuration of the synthesis module.

The configuration and setup of the synthesis module for the automated synthesis of [^68^Ga]Ga-DOTA-MGS8 is described in the Materials and Methods section (Section 4.2). The scheme of the synthesis module is shown in Figure 2.

Simultaneous elution of two Galli Ad generators was used for the synthesis of [^68^Ga]Ga-DOTA-MGS8. A starting activity of 1769 ± 125 MBq (*n* = 5) was calculated based on the specified elution yield of 60% of the available activity of gallium-68 at equilibrium, which was monitored by the weekly elution of the generators. Five batches of [^68^Ga]Ga-DOTA-MGS8 were produced with a total activity of 909 ± 150 MBq of the final product, corresponding to an average, decay-corrected radiochemical yield of 51.7 ± 10.9%, based on the eluted activity of gallium-68 and a total synthesis time of 39 min.

The final product met the specifications for radionuclide and [^68^Ga]Ga-DOTA-MGS8 identity. The percentage of the peak corresponding to [^68^Ga]Ga-DOTA-MGS8 determined by radio-HPLC was 92.8 ± 0.6% (*T*). Radionuclide incorporation based on radio-iTLC measurements was 99.4 ± 0.2% (*A*). In the Ph. Eur. monograph 2482, a formula for the calculation of the radiochemical purity is described, which considers gallium-68 in colloidal form determined by radio-iTLC and [^68^Ga]Ga-DOTA-MGS8 determined by HPLC. Based on the formula $RCP=A\times \left(\frac{T}{100}\right)$ a radiochemical purity of 92.2 ± 0.8% was calculated for the final product. The percentage of free gallium-68 determined by HPLC was 0.5 ± 0.2%. Radiochemical impurities related to radiolytic side products were detectable at a retention time of >6 min. For the determination of these radiochemical impurities, a relative retention time (RRT) of 0.45–0.95 and 1.05–1.20 and a limit of ≤8% was set. The peptide content in the final preparation was <40 µg. Table 1 summarizes specifications and the pre-release results obtained for each batch.

Three batches were additionally tested after the release for the ethanol and HEPES content, radionuclidic purity, as well as bacterial endotoxins and sterility (see Table 2).

**Figure 3:**
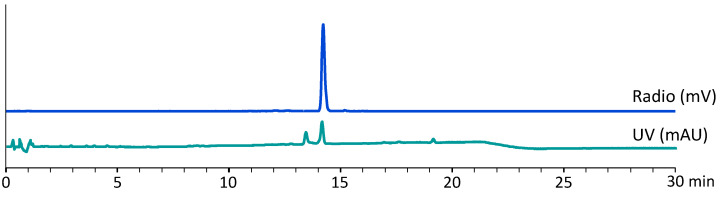
Representative radio-HPLC and UV-chromatogram of [^68^Ga]Ga-DOTA-MGS8.

In Figure 3, an exemplary radio-HPLC and UV chromatogram of the final product is shown.

### 2.2. Cell Internalization Studies

**Figure 4:**
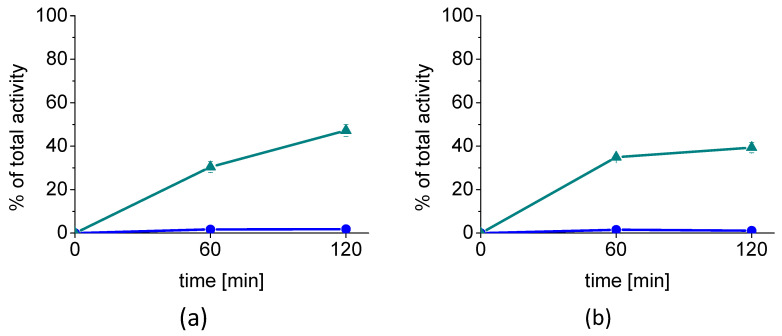
Cell internalization of [^68^Ga]Ga-DOTA-MGS8 in (**a**) A431-CCK2R cells and (**b**) AR42J cells after 1 and 2 h incubation (*n* = 3) (▲ internalized, ● membrane-bound).

Cell internalization studies carried out with two different cell lines confirmed a high receptor-mediated uptake of [^68^Ga]Ga-DOTA-MGS8. In the internalization studies using A431-CCK2R cells stably transfected to express human CCK2R, an internalized fraction of 30.4 ± 2.4% was observed after 1 h, which further increased to 47.2 ± 2.7%, after 2 h. The membrane-bound fraction with values of 1.7 ± 0.2% and 1.8 ± 0.07% at 1 and 2 h after incubation, respectively, was very low. Treatment with 1 µM pentagastrin resulted in blockage of the specific receptor interaction. Under these conditions, only an internalized fraction of 0.6 ± 0.2% and 1.0 ± 0.6% was found at 1 and 2 h, respectively. High cell internalization was also observed in AR42J cells with uptake values of 34.9 ± 1.0% and 39.4 ± 2.4% at 1 and 2 h after incubation, respectively. Under the same blocking conditions, the value for the internalized fraction was reduced to 0.2 ± 0.04% and 0.4 ± 0.1% for the same time points. Similar to A431-CCK2R cells, the membrane-bound fraction was also negligible, with only 1.5 ± 0.2% and 1.1 ± 0.3% for the two time points. The results of the cell internalization studies in A431-CCK2R and AR42J cells are summarized in Figure 4.

**Figure 5:**
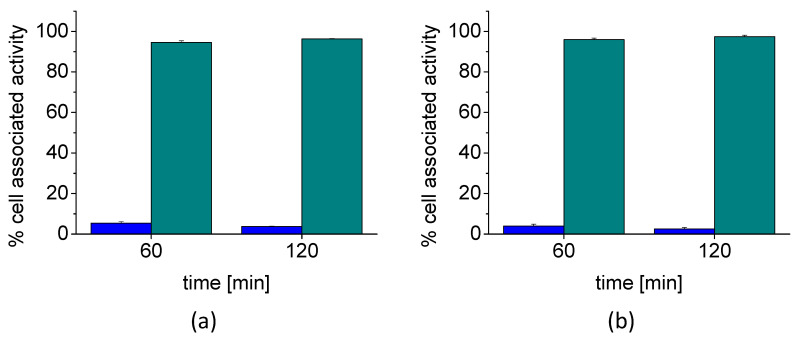
Distribution of the cell-associated radioactivity (internalized plus membrane-bound fraction) of [^68^Ga]Ga-DOTA-MGS8 in (**a**) A431-CCK2R cells and (**b**) AR42J cells after 1 and 2 h incubation (*n* = 3): membrane-bound (blue), internalized (cyan).

When considering the cell-associated radioactivity (internalized plus membrane-bound fraction), more than 94–96% of the radioligand was internalized by A431-CCK2R cells and 96–97% by AR42J cells in agreement with an agonist-induced receptor internalization (see Figure 5).

### 2.3. Biodistribution in BALB/c Nude Mice Bearing A431-CCK2R/A431-Mock Xenografts

Biodistribution studies in A431-CCK2R/A431-mock xenografted female BALB/c nude mice for the time point 1 h post-injection (p.i.) were performed using ^68^Ga-labeled DOTA-MGS8 at an injected radioactivity of ~200 kBq and ~30 pmol of total peptide. A favorable biodistribution profile was observed with the majority of the radioactivity (78.8 ± 3.6%) already excreted from the body within 60 min after injection.

**Figure 6:**
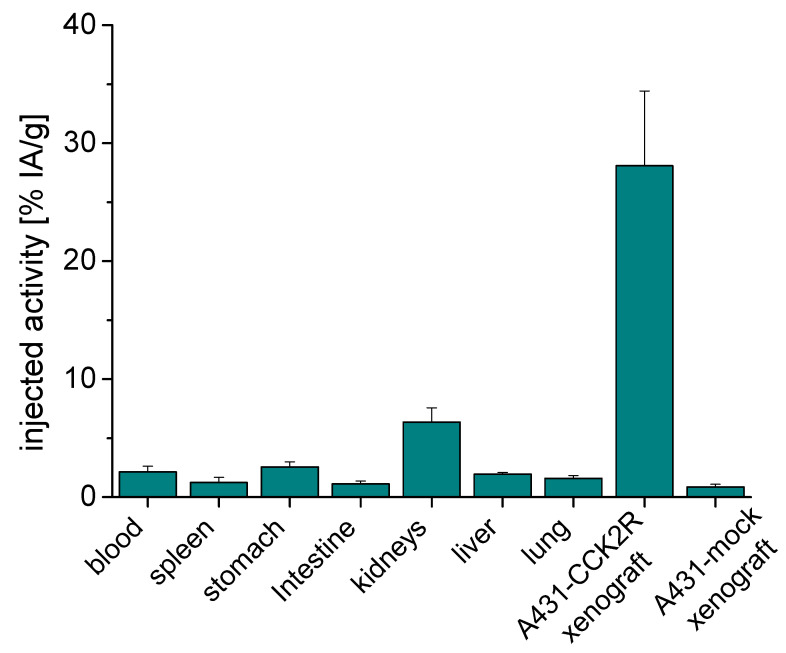
Biodistribution profile of [^68^Ga]Ga-DOTA-MGS8 in A431-CCK2R/A431-mock xenografted female BALB/c nude mice at 1 h p.i. (*n* = 4). Values are expressed as % IA/g.

For the analyzed time point of 1 h p.i., the highest accumulation of radioactivity in non-tumor tissues was observed for kidneys with an uptake value of 6.36 ± 1.21% IA/g, as the main route of excretion at the early time point. A much lower uptake was observed in the liver (1.94 ± 0.13% IA/g) and intestine (1.12 ± 0.23% IA/g). Further hepatic clearance may occur at a later time point after injection. The radioactivity in blood was 2.06 ± 0.46% IA/g, and consequently, slightly elevated values were also observed for highly perfused organs such as the lung (1.58 ± 0.25% IA/g) and spleen (1.22 ± 0.45% IA/g). Increased levels of radioactivity were further observed in CCK2R expressing stomach (2.56 ± 0.41% IA/g) as well as the pancreas (1.03 ± 0.15% IA/g). In all other tissues, the uptake levels were below 1%. At dissection, a mean weight of 117.8 ± 52.8 mg (range from 62.0 to 189.3 mg) was found for A431-CCK2R tumor xenografts. A similar value was also observed for A431-mock tumors (125.4 ± 67.3 mg; range from 59.9 to 219.2 mg). A high level of radioactivity was found in A431-CCK2R tumor tissue with uptake values of 28.08 ± 6.35% IA/g, resulting in a tumor-to-kidney ratio of 4.41 ± 0.40, a tumor-to-stomach ratio of 11.03 ± 2.05 and tumor-to-blood ratio of 13.38 ± 1.77. A very low non-specific accumulation was observed in A431-mock xenografts (0.86 ± 0.22% IA/g). Figure 6 and Table S1 summarize the tumor and organ uptakes. In Table 3, the tumor-to-normal tissue ratios are given for selected tissues.

## 3. Discussion

Radiolabeled peptides to be used in the diagnosis and therapy of various malignancies need to fulfill certain requirements such as robust and reproducible labeling with radioisotopes, specific targeting of receptors and high resistance against enzymatic catabolism [10]. Within the last years, the development of a radiolabeled gastrin derivative fulfilling this criterion has attracted increasing attention [10,12,15,19,20]. Among various tumor entities that can be considered for CCK2R targeting, patients with advanced MTC could especially benefit from this new diagnostic and therapeutic approach.

Different strategies for molecular design have been investigated, including but not limited to the substitution of selected amino acids (mainly replacement of methionine) or stereochemical inversion using D-amino acids (focused on the penta-Glu motif). These modifications allowed the limitations of in vivo stability and unfavorable tumor to non-target ratios to be overcome, especially with regard to high kidney uptake [21,22,23,24,25,26]. A considerable improvement leading to enhanced tumor-targeting properties was achieved by the introduction of specific modifications within the C-terminal receptor-specific binding sequence. The recently developed peptide analog DOTA-MGS5 was derived from the truncated minigastrin analog DGlu-Ala-Tyr-Gly-Trp-Met-Asp-Phe-NH_2_ (MG11) by a combined replacement of methionine in position 6 by *N*-methylated norleucine ((*N*-Me)Nle) and phenylalanine in position 8 by 1-naphthylalanine (1Nal). These modifications resulted in a noticeable benefit of in vivo stability, with reduced renal and elevated tumor uptake [15]. The safety of administration and diagnostic performance of [^68^Ga]Ga-DOTA-MGS5 PET/CT is currently being investigated in a pilot study at our center in patients with advanced MTC and other neuroendocrine tumors (EudraCT Number: 2020-003932-26). We recently reported the automated preparation and non-clinical studies required for clinical translation [27]. The first clinical application of [^68^Ga]Ga-DOTA-MGS5 in a 75-year-old female patient with recurrent MTC revealed liver lesions that were not detected by a prior [^18^F]FDOPA-PET/CT scan [28]. This highlights the benefit of the new PET imaging modality in the diagnostic work-up of patients with CCK2R-expressing tumor entities.

In recent studies by our group, further stabilization strategies within the N-terminal region of the peptide were investigated. Within the lead structure of DOTA-MGS5, the *N*-terminal amino acids in positions 2–4 were replaced by the cyclic amino acid proline [16]. This amino acid substitution introduces a tertiary amide bond within the peptide chain in analogy to *N*-methylated amide bonds or 1,4-disubstituted 1,2,3-triazoles, which could additionally promote the stability against enzymatic degradation [13]. Among three different peptide derivatives with proline substitution studied, DOTA-MGS8 radiolabeled with indium-111 and lutetium-177 showed the most promising advantages in terms of enhanced tumor uptake with concomitant low kidney uptake [16]. A stabilizing effect against enzymatic degradation in vivo could be observed, as demonstrated by the absence of the metabolites with cleavage at position DGlu-Pro, Pro-Tyr and Tyr-Gly in the blood and urine of mice intravenously injected with [^177^Lu]Lu-DOTA-MGS8 [29]. Thus, a considerable reduction of the enzymatic degradation compared to [^111^In]In-DOTA-MG11 could be achieved [11,24]. Comparably to [^177^Lu]Lu-DOTA-MGS5, ^111^In- and ^177^Lu-labeled DOTA-MGS8 revealed a high resistance against enzymatic degradation in metabolic studies in vivo with values of about 80% intact radiopeptide present in the blood of BALB/c mice 10 min after injection, leading to improved tumor targeting [15,16]. The achieved tumor uptake of [^177^Lu]Lu-DOTA-MGS8 and [^111^In]In-DOTA-MGS8 (~35% IA/g and ~43% IA/g) was superior to the improvement in tumor uptake, which was previously reported for [^111^In]In-DOTA-MG11 injected in combination with the neprilysin inhibitor phosphoramidon (~15% IA/g) [16,30]. Recently, also for non-peptidic CCK2R targeting antagonists, high tumor uptake was reported; however, this was combined with high renal retention [31,32]. Treatment with the mTORC1 inhibitor RAD001 (everolimus) also bears the potential to enhance CCK2R overexpression leading to improved tumor uptake of [^177^Lu]Lu-PP-F11N in A431-CCK2R xenografts (~20% IA/g), while simultaneously maintaining the uptake in other CCK2R-expressing organs unchanged [33]. Given the promising tumor-targeting properties reported for DOTA-MGS8 labeled with indium-111 and lutetium-177, in this study, we have evaluated the diagnostic properties of DOTA-MGS8 when radiolabeled with the positron-emitting radionuclide gallium-68.

The automated radiolabeling process based on a modified Scintomics GRP module connected to two Galli Ad ^68^Ge/^68^Ga generators described in this work for [^68^Ga]Ga-DOTA-MGS8 will support the clinical application of ^68^Ga-labeled stabilized minigastrin analogs in other nuclear medicine departments working with synthesis modules from different commercial suppliers. In the future, similar to the commercially available labeling kits for the simplified preparation of ^68^Ga-labeled somatostatin analogs and PSMA ligands, the kit-based preparation of a ^68^Ga-labeled minigastrin analog should be envisaged [34,35]. Given the rarity of a disease such as MTC, the widespread availability of the radiopharmaceutical is a key factor in simplifying the implementation of the evaluation of the CCK2R expression status in patients by PET/CT imaging also in other institutes. Using the described process, which was adopted from the routine production of other ^68^Ga-labeled radiopharmaceuticals and not specifically optimized for the production of [^68^Ga]Ga-DOTA-MGS8, a radiochemical yield >50% and a high radiochemical purity >91% could be achieved, suitable for possible clinical use.

The CCK2R targeting properties of ^68^Ga-labeled DOTA-MGS8 were evaluated in A431-CCK2R cells, stably transfected to express human CCK2R, as well as AR42J rat pancreatic cells, naturally expressing rat CCK2R. The introduction of proline into the N-terminal part of the linear peptide sequence had no apparent adverse influence on the cell uptake. The observed cell internalization of 47% after 2 h incubation in A431-CCK2R cells is in agreement with previous results observed for DOTA-MGS5 labeled with different radiometals (gallium-68: ~54%, indium-111: ~55%; lutetium-177: ~59%) as well as [^111^In]In-DOTA-MGS8 (~56%) studied in the same cell line [15]. In AR42J cells with physiological receptor expression, only a slightly decreased cell internalization of 35% to 39% after 1 and 2 h of incubation was observed, confirming the high receptor-specific cell uptake. Biodistribution studies of [^68^Ga]Ga-DOTA-MGS8 in the A431-CCK2R/A431-mock tumor-mouse model at the time point of 1 h p.i. showed a blood pool activity of 2.12 ± 0.51% IA/g and a kidney uptake of 6.36 ± 1.21% IA/g. Similar results were previously reported also for [^68^Ga]Ga-DOTA-MGS5 (blood: 1.47 ± 0.82%; kidney: 5.71 ± 1.38% IA/g) [15]. The uptake in tumor tissue, with values of 28.08 ± 6.35% IA/g, was only slightly increased by a factor of 1.2 in comparison with [^68^Ga]Ga-DOTA-MGS5 (23.25 ± 4.70% IA/g). Interestingly, the uptake of [^68^Ga]Ga-DOTA-MGS8 in the gastric wall, physiologically expressing CCK2R, was two times lower as compared to the reference (2.56 ± 0.41% vs. 5.12 ± 1.13% IA/g) [15]. A similar picture of the biodistribution profile was also observed for DOTA-MGS8 and DOTA-MGS5 labeled with indium-111 or lutetium-177. However, no clear statistically significant difference between the two peptide analogs could be found [15,16]. Thus, no conclusion on the superiority of DOTA-MGS8 over DOTA-MGS5 can be made. Both peptides display a similar in vivo stability and the introduction of proline in position 2 seems to have no considerable additional impact on the targeting potential. The tumor uptake of DOTA-MGS5 and DOTA-MGS8 is clearly superior to that of other minigastrin analogs such as [^177^Lu]Lu-DOTA-PP-F11N, which are currently being studied in clinical trials (ClinicalTrials.gov Identifier: NCT02088645) [12,15,16]. Radiopharmaceuticals based on stabilized minigastrin analogs, besides having confirmed applicability in PET/CT imaging, also show potential to improve the theranostic application in patients with CCK2R-expressing malignancies.

## 4. Materials and Methods

### 4.1. Materials

The precursor DOTA-MGS8 was obtained from piCHEM (Raaba-Grambach, Austria) in lyophilized aliquots of 1 mg. Sterile pharmaceutical formulations for parenteral application and reagents of Ph. Eur. grade or high purity for trace analysis were used for the automated synthesis. [^68^Ga]GaCl_3_ solution was obtained from two pharmaceutical-grade ^68^Ge/^68^Ga generators (Galli Ad, IRE EliT, Fleurus, Belgium), with a total activity of germanium-68 of 1850 MBq at the calibration date.

### 4.2. Radiolabeling

The automated synthesis of [^68^Ga]Ga-DOTA-MGS8 was performed using a Scintomics GRP4V module (Fürstenfeldbruck, Germany). The synthesis process was set up using a commercially available labeling kit consisting of a sterile disposable cassette for gallium-68 peptides (SC-01H) and a set of reagents (SC-01). A list of the valves, including all tubings and reagents of the cassette, is summarized in Table S2. An additional five-valve bank (IND-0015, ABX, Radeberg, Germany) was used for the gas supply (green line, MFC) and the connection to the vacuum sensor (blue line, valve 17 to the VAC sensor) needed for the automated elution process of the two ^68^Ge/^68^Ga generators. The two Galli Ad ^68^Ge/^68^Ga generators (Ire Elit, Fleurus, Belgium) were connected to the cassette (red line, valve 7) with three extension tubes (Lectro-Cath 1155.05, Vygon, Ecouen, France) and a BD Connecta three-way valve (394600, Becton Dickinson Infusion Therapy AB, Helsingborg, Sweden).

DOTA-MGS8 (1 mg) was diluted in 1 mL phosphate-buffered saline (PBS), aliquoted in fractions of 40 µg in reactor vials (ABX, Radeberg, Germany) and stored at −20 °C until use. Before starting the automated synthesis, DOTA-MGS8 was diluted in 2.5 mL of 1.5 M HEPES buffer and 100 µL of 6 M HCl, and the reactor vial was connected to the cassette (valve 2 and valve 12).

A 3 mL syringe containing a mixture of 1.5 mL of 5 M NaCl and 150 µL of 6 M HCl (valve 6), a 10 mL vial with 2.5 mL 50% ethanol (valve 9), a 250 mL water bag (valve 14), a 20 mL vial of PBS (valve 13) and a 10 mL vial with 5 mL 99.9% ethanol (valve 15) were mounted on the cassette.

The product vial was assembled with a 0.22 µm Cathivex^®^-GV low-protein-binding sterile filter (SLGV02505, Merck Millipore, Cork, Ireland) and a 0.2 µm Millex^®^ hydrophobic vent filter unit (SLGFN25V5, Merck, Darmstadt, Germany) and connected to the product line (valve 3).

The automatic synthesis started with the preconditioning of the Sep-Pak^®^ Light C18 cartridge (WAT023501, Waters, Milford, Massachusetts) connected to valve 4 and 5 with ethanol and water using the syringe pump. The elution process of the two Galli Ad generators was started by turning the rotary knob 90° for 20–30 s. The knob was moved to the “OFF” position again, followed by the transfer of the [^68^Ga]GaCl_3_ eluates using the vacuum pump of the module (VAC IN 1, connected to valve 1, horizontal port). A minimum negative pressure of 200 mbar was sufficient to transfer the eluates from the generators to the Chromafix^®^ PS-H^+^ SPE cartridge (731861, Marchery Nagel, Düren, Germany) connected to valve 1 (vertical port) and valve 6 (horizontal port). At the end of the transfer process (3 min), the vacuum was exhausted, and all further process steps were carried out using the syringe pump. [^68^Ga]GaCl_3_ was eluted from the PS-H^+^ SPE cartridge and transferred into the reaction vessel using 1.75 mL of the mixture of 1.5 mL of 5 M NaCl and 150 µL of 6 M HCl. This transfer step was carried out with the syringe pump by creating a vacuum in the reactor vial via valve 12. Radiolabeling was performed at 95 °C for 15 min. For purification, the reaction mixture was loaded onto the Sep-Pak^®^ C18 cartridge. After two washing steps with water to remove free gallium-68, the product was eluted with 2.5 mL of 50% EtOH into the sterile vial and diluted with ~15 mL PBS. The total synthesis time was 39 min.

### 4.3. Analytics

HPLC analysis of [^68^Ga]Ga-DOTA-MGS8 generated from the automated synthesis was performed using an Agilent 1260 series (Waldbronn, Germany) equipped with a DAD UV-detector (UV–VIS at λ = 280 nm) and a GABI Star radiometric detector (Elysia-Raytest, Straubenhardt, Germany). An ACE 3 C18 150 × 3 mm column (ACE111-1503, Advanced Chromatography Technologies, Aberdeen, UK) was used together with a flow rate of 0.42 mL/min and gradient elution with water/0.1% trifluoroacetic acid (TFA; A) and acetonitrile/0.1% TFA (B): 0–3 min 28% B, 3–15 min 28–50% B, 15–18 min 50% B, 1–19 min 50–28% B. For determining the identity of [^68^Ga]Ga-DOTA-MGS8, unlabeled DOTA-MGS8 (R_t_ 15.2 min) was used as a reference standard. From the radiochromatogram, the percentage of the peak corresponding to [^68^Ga]Ga-DOTA-MGS8 in relation to the total radioactivity was calculated. For this purpose, all peaks related to radioactivity were integrated by baseline-to-baseline integration and a RRT of 0.95–1.05 and a limit of ≥92% was considered for the peak corresponding to [^68^Ga]Ga-DOTA-MGS8. The peptide content was calculated from the UV chromatogram by baseline-to-baseline integration of the peptide-related peaks with RRT 0.8–1.2 in relation to DOTA-MGS8. For the peptide content, the sum of the peak areas corresponding to DOTA-MGS8 and metal complexes thereof observed in the UV trace was considered. The limit test for the peptide content was performed based on a calibration curve.

Radio-iTLC analysis was performed with a miniGITA Dual Head radio-iTLC scanner (Elysia-Raytest, Straubenhardt, Germany) using iTLC-SG strips (SGI0001, Agilent, Waldbronn, Germany) and a solvent mixture of 1 M ammonium acetate and methanol (1:1 *v/v*) as mobile phase. The radioactivity with a retardation factor (Rf) of 0.8–1.0 was considered to calculate the percentage of radionuclide incorporation, corresponding to the percentage of radioactivity due to complexed gallium-68 species.

A formula adopted from the Ph. Eur. monograph “2482” was used to calculate the radiochemical purity of [^68^Ga]Ga-DOTA-MGS8: $RCP=A\times \left(\frac{T}{100}\right)$, where *A* is the percentage radionuclide incorporation determined by iTLC and *T* corresponds to the percentage of the [^68^Ga]Ga-DOTA-MGS8 peak determined by radio-HPLC.

For the measurement of the total radioactivity and determination of the half-life, a dose calibrator was used (Isomed 2010, NuivaTech Healthcare, Paris, France). The radionuclide identity was additionally confirmed by gamma-ray spectrometry (ORTEC digiBASE, Tennesee, USA).

The pH of the final injectable solution was determined by the use of pH indicator strips (90417, Pehanon, Macherey-Nagel GmbH & Co. KG, Düren, Germany).

The ethanol content of the samples was determined by an accredited testing laboratory (Seibersdorf Laboratories, Seibersdorf, Austria).

A limit test for the HEPES content was performed using a validated HPLC method based on a 150 × 4.6 mm XBridge C18 5 µm column (186003116, Waters, Milford, Massachusets) and 20 mM solution of ammonium formate (pH 8) as mobile phase together with a flow rate of 0.7 mL/min modified from Antunes et al. [36]. The HEPES reference solution (40 µg/mL) and the sample were measured at UV λ = 195 nm.

Sterility testing, according to Ph. Eur. was performed post release.

Bacterial endotoxin testing was performed in 96-well apyrogenic microplates (3596, Costar, Corning Incorporated, New York, NY, USA) using a quantitative kinetic chromogenic Limulus Amebocyte Lysate assay (Kinetic-QCL 192 Test kit, 50-650U, Lonza, MD, USA). The measurements were performed with a FLUOstar Omega Microplate reader (BMG Labtech GmbH, Ortenberg, Germany) at UV λ = 405 nm [37].

### 4.4. Cell Internalization Study

Cell internalization studies were carried out using human A431 epidermoid carcinoma cells stably transfected with the plasmid pCR3.1, which comprises the complete coding sequence for the human CCK2R (A431-CCK2R) as well as AR42J rat pancreatic cell physiological expressing rat CCK2R.

For internalization studies, A431-CCK2R and AR42J cells were seeded in six-well plates (1 × 10^6^ cells for A431-CCK2R and 1.5 × 10^6^ for AR42J cells per well in DMEM or RPMI medium supplied with 10% (*v/v*) fetal bovine serum (FBS) and 1% penicillin/streptomycin/glutamine solution, respectively) 2 days prior to the assay. On the day of the cell internalization study, the incubation medium was discarded. The cells were rinsed with fresh medium and 1.2 mL DMEM or RPMI with 1% (*v/v*) FBS was added into each well. Radiolabeling of DOTA-MGS8 with gallium-68 for cell internalization studies is described in the Supplementary Materials including also an exemplary radio-HPLC and iTLC chromatogram (Figures S1 and S2). The cells were incubated in triplicate with ~100,000 cpm of [^68^Ga]Ga-DOTA-MGS8 in 300 μL PBS/0.5% BSA, resulting in a total volume of 1.5 mL, reaching a final concentration of 0.4 nM total peptide in the assay. Unspecific binding was evaluated using blocking conditions by co-incubation with 1 µM pentagastrin. After incubation at 37 °C for 1 and 2 h, internalization was interrupted by transferring the incubation medium in a separate tube. The cells were treated with two washing steps with PBS/0.5% BSA, which were transferred into the same tubes (non-internalized fraction). An acid wash was performed by treating the cells with a glycine buffer (50 mM, pH 2.8, 0.1 M NaCl) for 5 min to remove any membrane-bound radioligand (membrane-bound fraction). Subsequently, the cells were lysed using 2 × 1 mL 1 M NaOH and collected (internalized fraction). The fractions were measured together with a standard in an automatic gamma-counter (2480 Wizard2 3”, PerkinElmer Life Sciences and Analytical Sciences, formerly Wallac Oy, Turku, Finland). The percentage of radioactivity in the different fractions collected was evaluated by comparison with the standard representing the total radioactivity per well.

### 4.5. Biodistribution in BALB/c Nude Mice Bearing A431-CCK2R/A431-Mock Xenografts

Biodistribution studies were performed in accordance with the ethical standards of the institution and approved by the Austrian Ministry of Science (BMWFW-66.011/0072-V/3b/2019). In vivo studies were carried out in 6–8-week-old female BALB/c nude mice xenografted with A431-CCK2R/A431-mock cells (Charles River, Sulzfeld, Germany; *n* = 4). For the induction of the tumor xenografts, 150 μL DMEM containing 2 × 10^6^ A431-CCK2R and A431-mock cells were injected subcutaneously into the right and left flanks of each mouse. Tumors were allowed to grow for 10–14 days. [^68^Ga]Ga-DOTA-MGS8 used in animal studies was prepared as described in the Supplementary Materials (page 2, Figures S1 and S2). To remove any hydrophilic impurities after radiolabeling, the solution was passed through a Sep-Pak^®^ Light C18 cartridge (Waters, Milford, MA) preconditioned with ethanol and physiological saline. [^68^Ga]Ga-DOTA-MGS8 was then eluted with 600 µL of a mixture of ethanol and PBS (2/1; *v/v*) followed by 1100 µL PBS. To this solution, 20 µL of 0.01 M bicarbonate was added for pH adjustment. The final injection solution was diluted with PBS containing 0.5% bovine serum albumin (BSA) to reach an injected radioactivity of ~200 kBq and a total injected peptide amount of ~30 pmol. [^68^Ga]Ga-DOTA-MGS8 was administered in 150 µL via a lateral tail vein. The mice were euthanized 1 h p.i. by cervical spine dislocation. The blood was immediately drawn after sacrifice, and the organs of interest and the tumor tissues were dissected. All organs, including blood and the remaining body, were weighed and their radioactivity was measured using the gamma-counter together with a standard. For the determination of radioactivity in the different tissues, a standard of 10 µL of the injection solution, as well as a 50 µL aliquot of a 1:10 and 1:50 dilution of the injection solution, were measured along with samples taken from the animals. To improve accuracy, pipetted volumes were confirmed by weight. The volume injected to each mouse was determined by calculating the difference in weight of the syringe before and after injection. Results were expressed as the percentage of injected activity per gram tissue (% IA/g).

## 5. Conclusions

CCK2R targeting of radiolabeled MG analogs can be considerably improved by applying specific amino acid substitutions within the C-terminal receptor-specific binding site. The additional introduction of the cyclic amino acid proline within the N-terminal part of the peptide as described herein may contribute to the overall in vivo stability of the radiolabeled peptide but seems to have no significant impact on the targeting profile. The standardized, automated production using cassette-based synthesis modules already used in routine clinical practice and for the production of other radiopharmaceuticals also will facilitate the increased clinical translation of this new class of ^68^Ga-labeled MG analogs. [^68^Ga]Ga-DOTA-MGS8, comparably to [^68^Ga]Ga-DOTA-MGS5, has promising diagnostic properties for high-sensitivity PET/CT imaging.

## Figures and Tables

**Table 1:** Pre-release analyses of five batches of [^68^Ga]Ga-DOTA-MGS8.

Quality Control	Method	Criteria	Results (*n* = 5)
Appearance	Visual inspection	Clear, colorless solution, free of visible particles	Conforms
pH value	pH indicator strip	4–8	7
Volume	Graduated vial	15–20 mL	16–17 mL
Radioactivity concentration	Dose calibrator	≥22.2 MBq/mL	37.9 ± 8.7 MBq/mL
Radionuclide identity	Gamma-ray spectrometry	511 keV; 1022 keV	Conforms
Radionuclide identity	Half-life	62–74 min	68.2 ± 1.0 min
Identity of [^68^Ga]Ga-DOTA-MGS8 (comparison with reference)	HPLC	14.4–16.0 min	14.9 ± 0.1 min
Percentage of free gallium-68 (RRT 0.1–0.3)	Radio-HPLC	≤2%	0.5 ± 0.2%
Radiochemical impurities with RRT 0.45–0.95 and 1.05–1.20	Radio-HPLC	≤8%	6.7 ± 0.5%
Percentage of [^68^Ga]Ga-DOTA-MGS8	Radio-HPLC (*T*)	>92%	92.8 ± 0.6%
Radionuclide incorporation (retardation factor >0.8)	Radio-iTLC (*A*)	≥97%	99.4 ± 0.2%
Radiochemical purity	RCP = *A* × (*T*/100)	≥91%	92.2 ± 0.8%
DOTA-MGS8, [^68^Ga]Ga-DOTA-MGS8 and related substances (RRT 0.6–1.4)	HPLC	≤50 µg/V	38.9 ± 4.7 µg/V

**Table 2:** Post-release analyses of three batches of [^68^Ga]Ga-DOTA-MGS8.

Quality Control	Method	Criteria	Results (*n* = 3)
Ethanol content	Gas chromatography	≤10% (*v*/*v*)	Conforms
HEPES content	HPLC	≤500 µg/V	≤20
Radionuclide purity	Gamma-ray spectrometry	Ge-68 ≤ 0.001% (after >48 h)	Conforms
Bacterial endotoxins	LAL test	≤175 IU/V	≤40
Sterility	Ph. Eur.	Sterile	Conforms

**Table 3:** Tumor-to-normal tissue ratios for [^68^Ga]Ga-DOTA-MGS8 in A431-CCK2R xenografted female BALB/c nude mice at 1 h p.i. (*n* = 4).

Tumor-to-Normal Tissue Ratio	
Tumor-to-blood	13.38 ± 1.77
Tumor-to-kidney	4.41 ± 0.40
Tumor-to-stomach	11.03 ± 2.05

## Data Availability

Data are contained within the article and Supplementary Materials.

## Supplementary Materials

The following supporting information can be downloaded online; Figure S1: Radio-HPLC chromatogram of [^68^Ga]Ga-DOTA-MGS8 used for preclinical testing; Figure S2: Exemplary iTLC chromatogram of [^68^Ga]Ga-DOTA-MGS8 used for preclinical testing; Table S1: Uptake values in different organs of [^68^Ga]Ga-DOTA-MGS8 in A431-CCK2R/A431-mock xenografted female BALB/c nude mice at 1h p.i. (*n* = 4). Values expressed as % IA/g; Table S2: List of the valves of the cassette of the Scintomics GRP4V module, including tubings and reagents. V = vertical port, H = horizontal port.

## Sample Availability

Samples of the compound DOTA-MGS8 are available from the authors.
