# Supplementary material for: Automated Synthesis of 68Ga-Labeled DOTA-MGS8 and Preclinical Characterization of Cholecystokinin-2 Receptor Targeting
Source: Molecules. 2022 Mar 21;27(6):2034. doi: 10.3390/molecules27062034 (PMC8949806; doi:10.3390/molecules27062034)
Supplement: Supplementary file 1 [file molecules-27-02034-s001.zip › molecules-1557295-supplementary.pdf]

molecules

MDPI

Supplementary

Automated Synthesis of ^{68}Ga-Labeled DOTA-MGS8 and Preclinical Characterization of Cholecystokinin-2 Receptor Targeting

Anton Amadeus Hörmann [1,†], Elisabeth Plhak [2,3,†], Maximilian Klingler [1], Christine Rangger [1], Joachim Pfister [1], Gert Schwach [4], Herbert Kvaternik [3] and Elisabeth von Guggenberg [1,*]

Table of content

Supporting Materials and Results 1

Radiolabeling and Analytics of [^{68}Ga]Ga-DOTA-MGS8 for preclinical testing 1

Supporting Figures 3

Figure S1. Radio-HPLC chromatogram of [^{68}Ga]Ga-DOTA-MGS8 used for preclinical testing 3

Figure S2. Exemplary iTLC chromatogram of [^{68}Ga]Ga-DOTA-MGS8 used for preclinical testing 3

Supporting Tables 4

Table S1. Uptake values in different organs of [^{68}Ga]Ga-DOTA-MGS8 in A431-CCK2R/A431-mock xenografted female BALB/c nude mice at 1h p.i. (n=4). Values expressed as % IA/g. 4

Table S2. List and positions of the reagents of the Scintomics GRP4V Module. V = vertical port, H = horizontal port. 5

Supporting Materials and Results

Radiolabeling and Analytics of [^{68}Ga]Ga-DOTA-MGS8 for preclinical testing

Radiolabeling of DOTA-MGS8 with gallium-68 for the preclinical evaluation was performed manually with ~7.5 µg precursor and ~160 MBq of gallium-68. [^{68}Ga]$GaCl_3$ eluate was eluted from a commercial ^{68}Ge/^{68}Ga generator (IGG100 generator Eckert & Ziegler Radiopharma GmbH, Berlin, Germany) with 8 mL sterile ultrapure 0.1 M HCl solution (Rotem Industries Ltd., Beer-Sheva, Israel) with maximal theoretical activity of 1850 MBq. The gallium-68 was trapped on a SCX cartridge and eluted with 500 µL of 4.9 M NaCl/0.14 M HCl (1 GBq/mL). 15 µL of DOTA-MGS8 solution dissolved in PBS (0.5 µg/mL) were mixed in a low protein Eppendorf tube® (Eppendorf AG, Hamburg, Germany) with 400 µL of a 2 M sodium acetate/acetate buffer containing ~50 µL ethanol. A fraction of 200 µL of the concentrated gallium-68 eluate was added and the reaction mixture was incubated at 95 °C for 10 min.

Quality control for [^{68}Ga]Ga-DOTA-MGS8 prepared for preclinical tests was performed using an UltiMate 3000 chromatography system (Dionex, Germering, Germany) equipped with a HPLC pump, an autosampler, a variable UV-detector (UV-VIS at λ = 280nm) and a radiodetector (GabiStar, Raytest, Straubenhardt, Germany). A Phenomenex Jupiter 4 µm Proteo 90 Å C12 column, 250 × 4.6 mm (00G-4396-E0, Phenomenex Ltd., Aschaffenburg, Germany) was used with a flow rate of 1 mL/min together with the following water/0.1% TFA (A) and ACN/0.1% TFA (B) gradient: 0–3 min 10% B, 3–18 min 10–55% B, 18–20 min 55–80% B, 20–21 min 80–10% B, 21–25 min 10% B. iTLC measurements were performed using Agilent iTLC-SG, 9 x 1 cm chromatography paper and a 1:1 mixture of 1 M ammonium acetate and methanol as mobile phase for the determination of radionuclide incorporation. The percentage of impurities migrating with a retardation factor less than 0.2 was determined using a Scan-RAM radio-TLC scanner equipped with a PS Plastic/PMT detector (LabLogic Systems, Sheffield, UK). In Figure S2, an exemplary iTLC chromatogram is shown. To determine radionuclide incorporation, radioactivity with a redardation factor of 0.8-1.0 was used.

Manual radiolabeling of DOTA-MGS8 with gallium-68 resulted in a radiochemical purity >91% with ~1.0% free [^{68}Ga]$GaCl_3$ based on radio-HPLC analysis. The iTLC analysis using 1M ammonium acetate and methanol (1:1, v/v) as mobile phase confirmed <1% formation of colloidal gallium-68 species. Figure S1 and S2 shows an exemplary radio-HPLC chromatogram and iTLC chromatogram of [^{68}Ga]Ga-DOTA-MGS8, respectively.

Supporting Figures

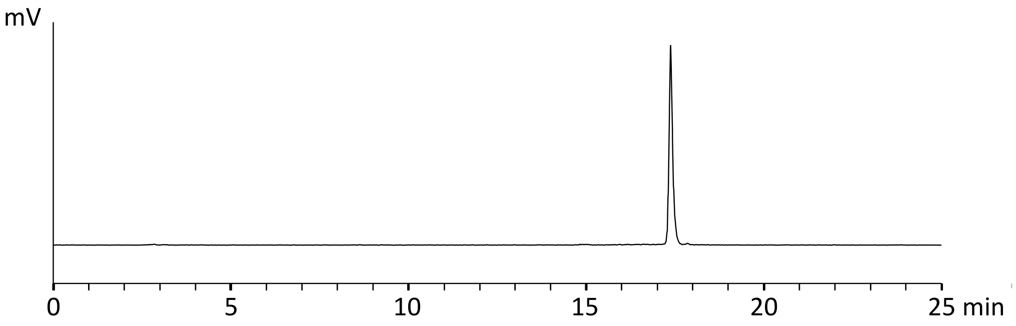

Figure S1. Radio-HPLC chromatogram of [^{68}Ga]Ga-DOTA-MGS8 used for preclinical testing

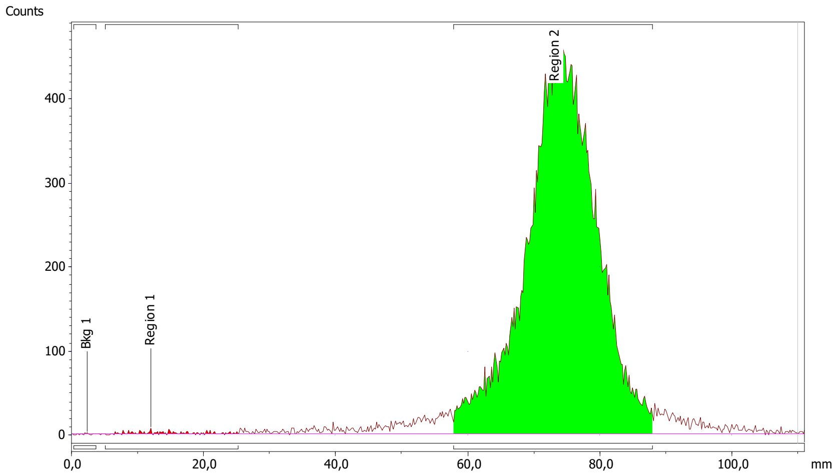

Figure S2. Exemplary iTLC chromatogram of [^{68}Ga]Ga-DOTA-MGS8 used for preclinical testing

Supporting Tables

Table S1. Uptake values in different organs of [^{68}Ga]Ga-DOTA-MGS8 in A431-CCK2R/A431-mock xenografted female BALB/c nude mice at 1h p.i. (n=4). Values expressed as % IA/g.

organ	mean ± SD
blood	2.12 ± 0.51
spleen	1.22 ± 0.45
pancreas	1.03 ± 0.15
stomach	2.56 ± 0.41
intestine	1.12 ± 0.23
kidneys	6.36 ± 1.21
liver	1.94 ± 0.13
heart	0.78 ± 0.07
lung	1.58 ± 0.25
muscle	0.45 ± 0.47
femur	0.58 ± 0.12
A431-CCK2R tumor	28.08 ± 6.35
A431-mock tumor	0.86 ± 0.22

Table S2. List of the valves of the cassette of the Scintomics GRP4V module, including tubings and reagents. V = vertical port, H = horizontal port.

Manifold Position	Materials/ Reagents	Further details
1 H	Connection to the waste bottle tube	Original part of the cassette
1 V	PSH+ cartridge with tubing at position 6 H	Original part of the cassette
2 V	Tubing to reaction vessel (main port)	Original part of the cassette
3 V	Tubing to product vial	25 mL evacuated vial, 22 µm Cathivex®-GV low protein binding sterile filter, 0.2 µm Millex®hydrophobic vent filter unit
4 V	Tubing to Sep-Pak C18 cartridge (outlet)	Original part of the cassette
5 V	Tubing to Sep-Pak C18 cartridge (inlet)	Original part of the cassette
5 H	Tubing to valve bench 2, valve 10 H	Original part of the cassette
6 H	Tubing to PSH+ cartridge	Original part of the cassette
6 V	1.5 M NaCl/ 6M HCl solution	3 mL syringe, original part of the cassette
7 V	Tubing lines to the ^{68}Ge/^{68}Ga Generators	3 x 50 cm lectrocath tubing, BD Connecta three-way valve
8 V	Syringe pump	20 mL syringe, original part of the cassette
9 V	White spike, 2.5 mL EtOH 99.9 %	10 mL Vial, original part of the cassette
10 H	Tubing to valve bench 1, valve 5 H	Original part of the cassette
10 V	Tubing to valve bench 3, valve 15 H	Original part of the cassette
11 H	Connection to the waste bottle tube	Original part of the cassette, position change from 11V to 11H
11 V	Tubing to valve bench 4, 16 H	10 cm
12 V	Tubing to reaction vessel (ventilation port)	Original part of the cassette
13 V	Green spike, 20 mL PBS (formulation buffer)	20 mL evacuated vial, original part of the cassette
14 V	Tubing to water bag spike	250 mL Water for injection
15 H	Tubing to valve bench 2, valve 10 V	Original part of the cassette
15 V	Brown spike, 5 mL EtOH 99.9 %	Original part of the cassette
16 V	Tubing to valve bench 3, 11 V	10 cm
16 H	Tubing to MFC sensor	35 cm, 0.2 µm Millex®hydrophobic vent filter unit
17 V	Tubing to vacuum sensor	35 cm
18 V	Closed	Combi Stopper (Braun, Melsungen, Germany)
19 V	Closed	Combi Stopper (Braun, Melsungen, Germany)
20 H	Vent	2 µm Millex®hydrophobic vent filter
20 V	Closed	Combi Stopper (Braun, Melsungen, Germany)
